# Medicare reimbursement for interventional pain procedures: 2000 to 2023

**DOI:** 10.1016/j.inpm.2024.100526

**Published:** 2024-11-30

**Authors:** Alexander M. Park, Aditya Khurana, Roger R. Wang, Adam E.M. Eltorai

**Affiliations:** a New York Medical College, Valhalla, NY, 10595, USA; b Mayo Clinic School of Medicine, Mayo Clinic, Scottsdale, AZ, 85259, USA; c Palo Pinto General Hospital, USA; d Brigham and Women's Hospital, Harvard Medical School, USA

**Keywords:** Interventional pain, Medicare, Reimbursement, Trends

## Abstract

**Background:**

An analysis of the financial trends of Interventional Pain (IP) procedures in the United States is lacking. Understanding these relations is necessary to help optimize future IP care delivery and costs.

**Objective:**

To examine Medicare reimbursement trends for IP procedures in both facility and non-facility settings.

**Methods:**

Utilizing the Physician Fee Schedule Look-Up Tool from the Centers for Medicare and Medicaid, reimbursement data for 32 of the most performed IP procedures was collected between 2000 and 2023 for both facility and non-facility clinical sites. After adjusting for inflation, annual change, total percent change, and compound annual growth rate (CAGR) were calculated for each procedure.

**Results:**

Following inflation adjustments, the average reimbursement rate decreased by an average of 61.31 % for facility procedures over the study period and by 60.40 % for non-facility procedures. The average adjusted reimbursement rate for facility procedures decreased by $6.76 per year with an average CAGR of −4.38 %, while the average adjusted reimbursement rate for non-facility procedures decreased by $18.66 per year with an average CAGR of −4.48 %. A two-tailed *t*-test was performed between facility and non-facility groups for total percent change (P = 0.803), annual change (*P* < 0.001), and CAGR (*P* = 0.746).

**Conclusion:**

Medicare reimbursement rates in both facility and non-facility settings have decreased from 2000 to 2023, with non-facility procedures experiencing a significantly larger decrease.

## 1 Introduction

As a relatively young field, Interventional Pain (IP) is constantly evolving and innovating. As the field and the scope of practice grows, understanding the economic underpinnings of the specialty, in both facility and non-facility settings, is becoming increasingly important. IP procedures, such as nerve blocks, epidural injections, spinal cord stimulation, and others require a deep understanding of technique and the complexity of a patient's pain [1,2]. These procedures can be easily offered in both non-facility and facility settings; therefore, physicians should be aware of the economic trends surrounding them. Facility services are provided within a hospital, ambulatory surgery center, or skilled nursing facility setting while non-facility services are provided everywhere else including outpatient clinics, urgent care centers, and home services. Previous studies have shown decreases in Medicare reimbursement trends in the fields of general surgery [3], orthopedics [4], radiation oncology [5], and diagnostic imaging [6]. To date, a comprehensive analysis of physician reimbursement trends in IP management is lacking. The objective of this investigation is to comprehensively evaluate reimbursement trends for common procedures in IP in different clinical settings.

## 2 Methods

A literature search was performed to determine the Current Procedural Terminology (CPT) codes for the top 32 most frequently performed IP procedures reported by Manchikanti et al. [7] (Table 1). The Physician Fee Schedule Look-Up Tool from the Centers for Medicare and Medicaid Services (CMS) was utilized to collect pricing information for each CPT code throughout the study period years 2000–2023. The average facility and non-facility price data was pulled for this study. The data for this study was extracted from a publicly available database on Medicare reimbursement schedules and was therefore exempt from an IRB.

**Table 1:** Top 32 interventional pain procedure CPT codes.

CPT	Abbreviation	Description
27093	IPHMR	Injection procedure for HIP arthrography - without anesthesia
62263	PCTEA2	Percutaneous epidural adhesiolysis - 2 or 3 days
62264	PCTEA1	Percutaneous epidural adhesiolysis - 1 day
62282	NELS	Neurolytic epidural, L/S
27096	IPSIJMR	(G0260) Injection procedure for sacroiliac joint, arthrography
62290	IPDL	Injection procedure for discography each level: lumbar
62291	IPDCT	Injection procedure for discography each level: C/T
62310 (2000–2016)	CEF	Cervical epidural w/and w/o fluoroscopy
62320 (2017–2023)		
62311 (2000–2016)	LEF	Lumbar epidural w/and w/o fluoroscopy
62322 (2017–2023)		
62318 (2000–2016)	ESACCT	Epidural or subarachnoid, catheterization, C/T
62324 (2017–2023)		
62319 (2000–2016)	CAELS	Catheterization, epidural, L/S
62326 (2017–2023)		
64400	TNI	Injection, anesthetic agent; trigeminal nerve, any division or branch
64405	GON	Greater occipital nerve
64418	SSN	Suprascapular nerve
64420	ICS	Intercostal, single
64421	ICM	Intercostal, multiple, regional block
64450	PNB	Other peripheral nerve or branch
64480	CTFESI	Cervical transforaminal epidural injections add-on
64483	LSTFESI	Lumbar/sacral transforaminal epidural injections
64484	LSTFESIA	Lumbar/sacral transforaminal epidural injections add-on
64470 (2000–2009)	CTFJI1	Cervical and thoracic facet joint injections 1st Level (Id 64470)
64490 (2010–2023)		
64472 (2000–2009)	CTFJI2	Cervical and thoracic facet joint injections 2nd Level (Id 64472)
64491 (2010–2023)		
64472 (2000–2009)	CTFJI3	Cervical and thoracic facet joint injections 3rd Level (Id 64472)
64492 (2010–2023)		
64475 (2000–2009)	PVFJNLS1	Paravertebral facet joint or facet joint nerve; lumbar/sacral, 1st Level (old 64475)
64493 (2010–2023)		
64476 (2000–2009)	PVFJNLS2	Paravertebral facet joint or facet joint nerve; lumbar/sacral, 2nd Level (old 64476)
64494 (2010–2023)		
64476 (2000–2009)	PVFJNLS3	Paravertebral facet joint or facet joint nerve; lumbar/sacral, 3rd Level (old 64476)
64495 (2010–2023)		
64622 (2000–2011)	DNLAPVFJNLSS	Destruction by neurolytic agent, paravertebral facet joint nerve; lumbar or sacral, single level
64633 (2012–2023)		
64623 (2000–2011)	DNLAPVFJNLSA	Destruction by neurolytic agent, paravertebral facet joint nerve; lumbar or sacral, each additional level
64634 (2012–2023)		
64626 (2000–2011)	DNLAPVFJNCTS	Destruction by neurolytic agent, paravertebral facet joint nerve; cervical or thoracic, single level
64635 (2012–2023)		
64627 (2000–2011)	DNLAPVFJNCTA	Destruction by neurolytic agent, paravertebral facet joint nerve; cervical or thoracic, each additional level
64636 (2012–2023)		
64640	CNLAPNB	Destruction by neurolytic agent; other peripheral nerve or branch
64680	DNLARMCP	Destruction by neurolytic agent, with or without radiologic monitoring; celiac plexus

Adapted from Manchikanti et al.

Schedules are calculated using Relative Value Units (RVU), which is a calculated combination related to physician work, resources, and expenses that go into each procedure [8]. The CMS will determine the final RVU, which is then multiplied by the annual conversion factor and adjusted according to geographic indices. The data from the Physician Fee Schedule Look-Up Tool was separated into “facility” and “non-facility” and averaged per procedure by CPT code per year for the duration of the study period. The CMS defines facility care as provided in hospitals, ambulatory surgery centers, skilled nursing homes/facilities and hospice facilities. Non-facility care can be considered anywhere else and can include outpatient clinics, urgent care clinics, and physician offices.

The American Medical Association (AMA) provides the CPT codes, which are the standard descriptive terminologies used for the most widely accepted medical procedural nomenclature used for billing and reimbursement processes [9]. This list of CPT codes is updated annually and therefore, previous changes for these codes were adjusted for through a method known as “crosswalking [10].” Certain CPT code procedures did not exist in earlier years and were crosswalked to find the old CPT codes that represented the same procedures. Although the code has changed, the procedure is the same for both the older and newer codes.

For each procedure in both facility and non-facility settings, the raw (unadjusted) percent change was calculated and averaged (Table 2a). This was compared to the percent change over the same period in the general consumer price index (CPI), which was acquired from the US Department of Labor Bureau of Labor Statistics [11] (Table 2b). A 2-tailed *t*-test was used for the comparison. The data was then adjusted for inflation [12] and the average adjusted percent change, adjusted compound annual growth rate (CAGR), and average annual change was calculated (Table 3). CAGR is a measure of annual growth rate over a given period and is often used to evaluate the performance of investments over time while accounting for the compounding effects of growth. CAGR was calculated with the adjusted 2023 data using the following formula [13]:

$$\text{CAGR}=\left[{\left(\frac{2023\;\text{Value}}{2000\;\text{Value}}\right)}^{\frac{1}{2023-2000}}\right]-1$$

**Table 2a:** Unadjusted (RAW) reimbursement trends.

Abbreviation	Average Reimbursement Rate 2000 (In 2000 Dollars)		Average Reimbursement Rate 2023 (In 2023 Dollars)		Average Raw Percent Change	
	Facility	Non-Facility	Facility	Non-Facility	Facility	Non-Facility
IPHMR	$114.40	$383.33	$69.82	$248.55	−38.97 %	−35.16 %
PCTEA2	$505.36	$641.79	$84.76	$170.58	−83.23 %	−73.42 %
PCTEA1∗	$316.82	$850.02	$326.54	$670.54	3.07 %	−21.11 %
NELS	$204.79	$338.41	$249.55	$460.91	21.86 %	36.20 %
IPSIJMR	$86.38	$625.36	$145.38	$330.50	68.30 %	−47.15 %
IPDL	$263.89	$354.85	$160.68	$368.50	−39.11 %	3.85 %
IPDCT	$253.26	$350.95	$148.83	$339.27	−41.24 %	−3.33 %
CEF	$141.82	$302.37	$102.51	$172.37	−27.72 %	−42.99 %
LEF	$117.84	$304.79	$81.96	$144.37	−30.45 %	−52.63 %
ESACCT	$153.41	$315.67	$90.96	$143.80	−40.71 %	−54.44 %
CAELS	$277.94	$614.80	$87.51	$144.96	−68.52 %	−76.42 %
TNI	$80.77	$140.27	$51.34	$117.30	−36.43 %	−16.37 %
GON	$98.01	$162.01	$53.47	$77.23	−45.44 %	−52.33 %
SSN	$99.19	$164.87	$56.83	$90.52	−42.71 %	−45.10 %
ICS	$96.48	$151.50	$59.81	$101.66	−38.00 %	−32.90 %
ICM	$134.40	$196.71	$24.86	$34.08	−81.50 %	−82.67 %
PNB	$92.49	$135.16	$42.84	$78.30	−53.68 %	−42.07 %
CTFESI	$112.00	$305.13	$62.42	$140.80	−44.27 %	−53.86 %
LSTFESI	$137.70	$312.30	$113.23	$258.28	−17.77 %	−17.30 %
LSTFESIA	$96.94	$288.39	$52.43	$116.98	−45.91 %	−59.44 %
CTFJI1	$135.42	$311.72	$107.27	$199.48	−20.79 %	−36.01 %
CTFJI2	$96.42	$261.48	$60.33	$100.76	−37.43 %	−61.47 %
CTFJI3	$96.42	$261.48	$61.39	$101.47	−36.33 %	−61.20 %
PVFJNLS1	$103.64	$277.13	$92.33	$184.54	−10.91 %	−33.41 %
PVFJNLS2	$71.88	$262.21	$52.08	$94.28	−27.55 %	−64.04 %
PVFJNLS3	$71.88	$262.21	$52.79	$94.28	−26.56 %	−64.04 %
DNLAPVFJNLSS	$253.23	$347.55	$195.69	$459.89	−22.72 %	32.32 %
DNLAPVFJNLSA	$90.96	$170.69	$68.00	$271.56	−25.25 %	59.10 %
DNLAPVFJNCTS	$245.23	$396.25	$196.04	$464.15	−20.06 %	17.14 %
DNLAPVFJNCTA	$86.95	$236.84	$60.04	$255.44	−30.95 %	7.85 %
CNLAPNB	$217.41	$315.10	$121.26	$258.50	−44.23 %	−17.96 %
DNLARMCP	$219.80	$272.02	$165.98	$365.29	−24.48 %	34.29 %
Average					−31.55 %	−29.88 %

∗ PCTEA1 (code 62264) data was not present 2000–2003 (2003 data used instead of 2000).

**Table 2b:** Average reimbursement percent change and CPI.

Unadjusted Percent Change in Consumer Price Index (2000–2023)	P Value of Comparison Between Average Reimbursement Percent Change and CPI	
	Facility	Non-Facility
76.95 %	p < 0.001	p < 0.05

**Table 3:** Adjusted reimbursement trends.

Abbreviation	Average Reimbursement Rate 2000 (In 2023 Dollars)		Average Reimbursement Rate 2023 (In 2023 Dollars)		Average Adjusted Percent Change (%)		Adjusted CAGR (%)		Annual Change ($)	
	Facility	Non-Facility	Facility	Non-Facility	Facility	Non-Facility	Facility	Non-Facility	Facility	Non-Facility
IPHMR	$203.06	$680.41	$69.82	$248.55	−65.62 %	−63.47 %	−4.54 %	−4.28 %	-$5.12	-$32.49
PCTEA2	$897.01	$1139.18	$84.76	$170.58	−90.55 %	−85.03 %	−9.75 %	−7.92 %	-$29.15	-$43.93
PCTEA1^a^	$526.24	$1411.88	$326.54	$670.54	−37.95 %	−52.51 %	−2.05 %	−3.19 %	-$9.77	-$25.78
NELS	$363.50	$600.68	$249.55	$460.91	−31.35 %	−23.27 %	−1.62 %	−1.14 %	-$5.75	-$22.60
IPSIJMR	$153.32	$1110.01	$145.38	$330.50	−5.18 %	−70.23 %	−0.23 %	−5.13 %	-$1.53	-$36.64
IPDL	$468.40	$629.86	$160.68	$368.50	−65.70 %	−41.49 %	−4.55 %	−2.30 %	-$11.51	-$20.61
IPDCT	$449.54	$622.94	$148.83	$339.27	−66.89 %	−45.54 %	−4.69 %	−2.61 %	-$10.83	-$18.84
CEF	$251.73	$536.71	$102.51	$172.37	−59.28 %	−67.88 %	−3.83 %	−4.82 %	-$5.77	-$19.11
LEF	$209.17	$541.00	$81.96	$144.37	−60.82 %	−73.31 %	−3.99 %	−5.58 %	-$4.67	-$19.62
ESACCT	$272.30	$560.31	$90.96	$143.80	−66.59 %	−74.34 %	−4.66 %	−5.74 %	-$7.28	-$23.57
CAELS	$493.34	$1091.27	$87.51	$144.96	−82.26 %	−86.72 %	−7.24 %	−8.40 %	-$8.72	-$26.25
TNI	$143.37	$248.98	$51.34	$117.30	−64.19 %	−52.89 %	−4.37 %	−3.22 %	-$3.40	-$7.28
GON	$173.97	$287.57	$53.47	$77.23	−69.26 %	−73.14 %	−5.00 %	−5.56 %	-$5.16	-$9.26
SSN	$176.06	$292.64	$56.83	$90.52	−67.72 %	−69.07 %	−4.80 %	−4.97 %	-$4.56	-$11.27
ICS	$171.25	$268.91	$59.81	$101.66	−65.07 %	−62.20 %	−4.47 %	−4.14 %	-$3.84	-$13.23
ICM	$238.56	$349.16	$24.86	$34.08	−89.58 %	−90.24 %	−9.36 %	−9.62 %	-$7.59	-$23.36
PNB	$164.17	$239.91	$42.84	$78.30	−73.91 %	−67.36 %	−5.67 %	−4.75 %	-$5.91	-$8.28
CTFESI	$198.80	$541.61	$62.42	$140.80	−68.60 %	−74.00 %	−4.91 %	−5.69 %	-$6.23	-$16.43
LSTFESI	$244.42	$554.33	$113.23	$258.28	−53.67 %	−53.41 %	−3.29 %	−3.27 %	-$5.42	-$22.76
LSTFESIA	$172.07	$511.89	$52.43	$116.98	−69.53 %	−77.15 %	−5.04 %	−6.22 %	-$5.43	-$17.89
CTFJI1	$240.37	$553.30	$107.27	$199.48	−55.37 %	−63.95 %	−3.45 %	−4.34 %	-$5.27	-$21.40
CTFJI2	$171.15	$464.13	$60.33	$100.76	−64.75 %	−78.29 %	−4.43 %	−6.43 %	-$4.46	-$14.89
CTFJI3	$171.15	$464.13	$61.39	$101.47	−64.13 %	−78.14 %	−4.36 %	−6.40 %	-$4.40	-$14.85
PVFJNLS1	$183.96	$491.91	$92.33	$184.54	−49.81 %	−62.48 %	−2.95 %	−4.17 %	-$3.50	-$18.92
PVFJNLS2	$127.59	$465.42	$52.08	$94.28	−59.18 %	−79.74 %	−3.82 %	−6.71 %	-$2.88	-$14.00
PVFJNLS23	$127.59	$465.42	$52.79	$94.28	−58.63 %	−79.74 %	−3.76 %	−6.71 %	-$2.83	-$14.00
DNLAPVFJNLSS	$449.48	$616.90	$195.69	$459.89	−56.46 %	−25.45 %	−3.55 %	−1.27 %	-$6.58	-$14.27
DNLAPVFJNLSSA	$161.45	$302.97	$68.00	$271.56	−57.88 %	−10.37 %	−3.69 %	−0.47 %	-$2.12	-$3.97
DNLAPVFJNCTS	$435.28	$703.34	$196.04	$464.15	−54.96 %	−34.01 %	−3.41 %	−1.79 %	-$9.79	-$16.36
DNLAPVFJNCTA	$154.34	$420.39	$60.04	$255.44	−61.10 %	−39.24 %	−4.02 %	−2.14 %	-$3.52	-$9.75
CNLAPNB	$385.90	$559.30	$121.26	$258.50	−68.58 %	−53.78 %	−4.91 %	−3.30 %	-$15.17	-$22.52
DNLARMCP	$390.15	$482.84	$165.98	$365.29	−57.46 %	−24.34 %	−3.65 %	−1.21 %	-$8.24	-$13.09
Average					−61.31 %	−60.40 %	−4.38 %	−4.48 %	-$6.76	-$18.66

All values adjusted for inflation.

a PCTEA1 (code 62264) data was not present 2000–2003 (2003 data used instead of 2000).

**Fig. 1:**
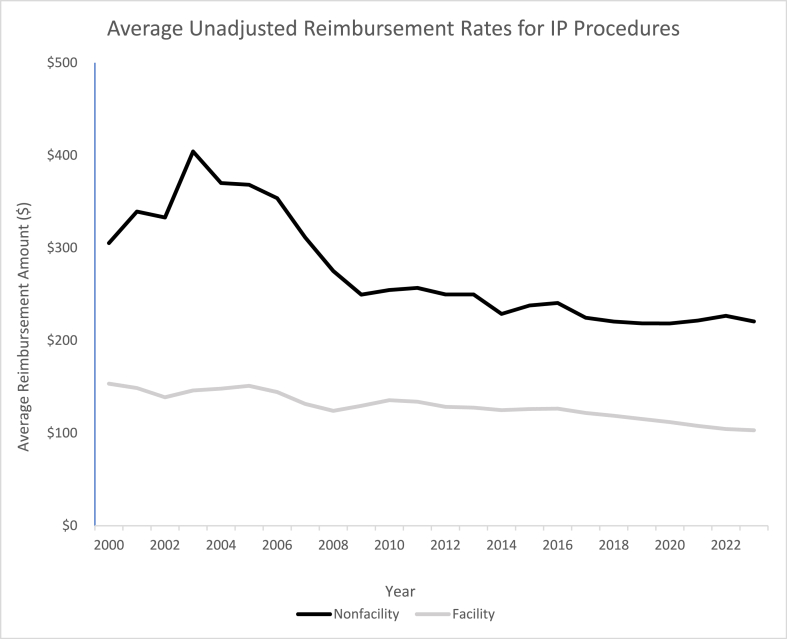
Average Unadjusted Reimbursement Rates for Top IP Procedures (Nonfacility bolded).

**Fig. 2:**
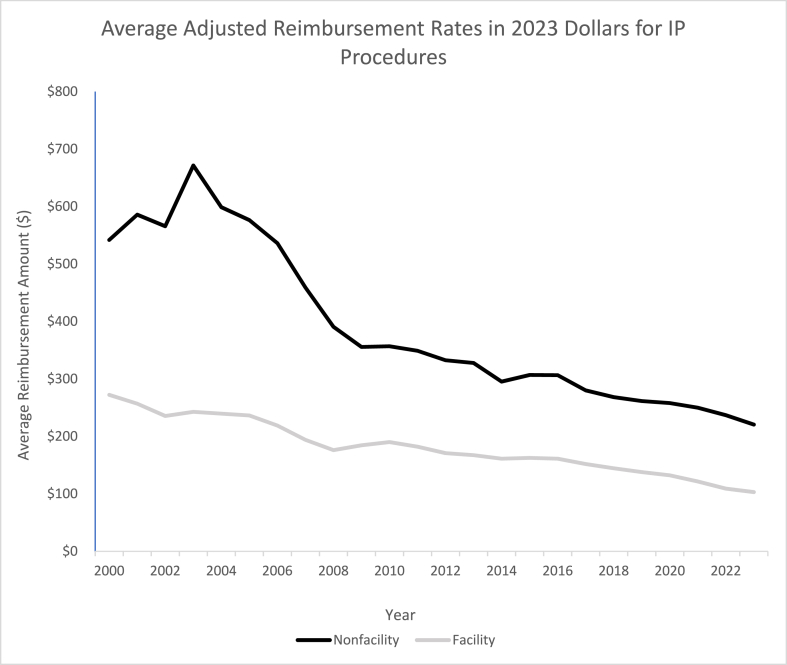
Average Adjusted Reimbursement Rates in 2023 US Dollars for Top IP Procedures (Nonfacility bolded).

A least squares linear regression was used to calculate the average annual change for each procedure. The raw and adjusted data in both settings were also analyzed by year for all IP Procedures (Table 4). The trends for average annual raw reimbursement rates by setting (Fig. 1) and average annual adjusted reimbursement rates by setting (Fig. 2) were also calculated and shown. Additional analysis was performed to compare the average adjusted percent change for all facility procedures and all non-facility procedures from 2000 to 2023 (Table 5). The average CAGR and annual change for both settings were also calculated and compared through a 2-tailed *t*-test. Institutional review board approval was not required for this study as all data used in this analysis is publicly available.

**Table 4:** Average annual reimbursement amount.

Average Annual Reimbursement Amount				
	Average Raw Reimbursement Amount ($)		Average Adjusted Reimbursement Amount ($)	
Year	Nonfacility	Facility	Nonfacility	Facility
2000	$305.27	$153.43	$541.85	$272.34
2001	$339.27	$148.77	$585.92	$256.93
2002	$332.91	$138.70	$565.61	$235.64
2003	$404.21	$146.12	$671.39	$242.71
2004	$370.13	$148.04	$598.87	$239.53
2005	$368.26	$151.12	$576.33	$236.50
2006	$353.71	$144.36	$536.22	$218.84
2007	$311.30	$131.58	$458.86	$193.95
2008	$275.13	$124.09	$390.68	$176.21
2009	$249.55	$129.57	$355.61	$184.63
2010	$254.56	$135.58	$356.89	$190.08
2011	$256.83	$133.94	$349.03	$182.02
2012	$249.80	$128.41	$332.48	$170.92
2013	$249.77	$127.54	$327.70	$167.33
2014	$228.79	$124.89	$295.37	$161.24
2015	$237.93	$126.07	$306.93	$162.63
2016	$240.62	$126.53	$306.55	$161.20
2017	$224.66	$121.78	$280.15	$151.86
2018	$220.51	$118.76	$268.36	$144.53
2019	$218.60	$115.33	$261.45	$137.94
2020	$218.48	$111.98	$258.02	$132.25
2021	$221.64	$107.85	$250.01	$121.65
2022	$226.68	$104.41	$236.88	$109.11
2023	$220.60	$103.09	$220.60	$103.09

**Table 5:** Average adjusted reimbursement trends.

Adjusted Percent Change (%)	CAGR (%)	Annual Change ($)
−61.31 %	−4.38 %	-$6.76
−60.40 %	−4.48 %	-$18.66
0.803	0.746	p < 0.001

All values adjusted for inflation.

## 3 Results

Throughout the duration of the study period, the average raw reimbursement rate in facility settings decreased by 31.55 % and by 29.88 % in non-facility settings (Table 2a). However, during the same period, the CPI increased by 76.95 % (Table 2b). When compared to both facility and non-facility data, the increase in CPI was significantly greater than the observed decreased change in reimbursement for the top IP procedures (P < 0.001 and P < 0.05, respectively). A representation of these trends can be seen for the raw and unadjusted data showing a noticeably steeper decrease in average non-facility reimbursements when compared to average facility reimbursements. (Fig. 1).

The data was then adjusted for inflation in 2023 dollars and showed a further decreasing trend in average reimbursement rates. The average reimbursement rate in facility settings decreased by 61.31 % and by 60.40 % in non-facility settings (Table 3). This is about a 30 % greater decrease than the unadjusted rates (−31.55 % and −29.88 %, respectively) seen in Table 2a. Over the study period, both the facility and non-facility setting procedures experienced a decrease in average CAGR (−4.38 % and −4.48 %, respectively) as well as a decrease in average annual change (-$6.76 and -$18.66, respectively). The only statistically significant downwards trend, however, was annual change (P < 0.001) showing that non-facility procedures were reimbursed at a significantly lower rate on an annual basis (Table 5). A representation of these trends analyzed by year can be seen for the adjusted data (Fig. 2), depicting a greater decrease in average nonfacility procedure reimbursement. The average raw and adjusted reimbursement amount by year can be seen in Table 4 showing a downwards trend.

## 4 Discussion

The purpose of this study was to evaluate the monetary trends of Medicare reimbursement patterns in IP procedures over the study period (2000–2023) and examine the difference in trends between facility and non-facility care. Since 2000, the utilization rate of IP procedures has and continues to increase [14]. Therefore, understanding how location and reimbursement interact to affect access to these procedures will be crucial for future physicians, administrators, and legislators in future years as the population in the US ages and more individuals join the Medicare program.

It is important to note that procedure utilization varies greatly by practice and physician preference, however steroid injections and facet procedures are performed by most IP providers. Cervical epidural steroid injection (64480) reimbursement decreased more (68.6 % and 74 %, facility and non-facility, respectively) than lumbar/sacral epidural steroid injections (64483) which decreased 53.67 % and 53.41 %, facility and non-facility, respectively. Paravertebral facet joint nerve destruction procedures experienced similar decreases ranging from 54.96 % to 68.58 % in facility settings, which was comparable to the average facility change of 61.31 %. There was greater variability of reimbursement in non-facility settings, ranging from 10.37 % to 39.24 %, which was less than the average non-facility change of 60.40 %. After inflation adjustment, percutaneous epidural adhesiolysis – 2 or 3 days (PCTEA2, 62263) and intercostal multiple region blocks (ICM,64421) were shown to have the largest adjusted percentage change in both facility and non-facility settings (Table 3). Reimbursement for PCTEA2 decreased 90.55 % in facility settings and 85.03 % in non-facility settings while reimbursement for ICM decreased 89.58 % in facility and 90.24 % in non-facility settings. Lumbar/sacral epidural catheterizations (CAELS, 62319/62326) also decreased by 82.26 % in facility and 867.72 % in non-facility settings. Some procedures, however were not as impacted by the decreasing reimbursements. In facility settings, sacroiliac joint arthrography injections (IPSIJMR, 27096) only experienced a 5.18 % decrease. In non-facility settings, additional cervical/thoracic level paravertebral facet joint destruction (DNLAPVFJNLSA, 64623/64634) only decreased by 10.37 %.

In our data analysis prior to adjustment for inflation, the average raw reimbursement rate decreased by a larger amount in facility procedures (31.55 %) than in non-facility procedures (29.88 %). Of note, the raw data showed three procedures in the facility setting (62264, 62282, 27096) and seven procedures in the non-facility setting (62282, 62290, 64633, 64634, 64635, 64636, and 64680) experience an increase in raw percent change (Table 2a). The average reimbursement rate also decreased by a larger amount after adjusting for inflation rate in both facility (61.31 %) and non-facility procedures (60.40 %) (Table 3). Importantly, although the data was adjusted for inflation, the conversion factor utilized in the determination of Medicare reimbursement schedules has not. General inflation has increased 78.3 % since 2000 [12], while the conversion rate has barely changed at all. This inconsistency is likely larger since medical inflation rates are higher than general inflation rates [15]. The overall downwards trend in Medicare reimbursements may also be attributed to the budget neutrality the CMS must maintain every year. The current law prevents the projected costs of total Medicare spending may not increase more than $20 million each year. With a greater volume of these procedures being performed, the CMS theoretically needs to reimburse at a higher cost. However, due to this budget neutrality they are not able to and therefore may decrease reimbursement per procedure. This leads to increased physician utilization and performance of these procedures, further contributing to a never-ending cycle [16].

A crucial factor in the understanding of Medicare reimbursements is that the RVU assigned to each procedure will vary by setting. The RVU assigned to non-facility procedures are usually higher because the services are delivered in “a setting where no other Medicare payment system covers the facility-related expenses [16].” In a hospital, or facility setting, costs for supplies and personnel are covered by the hospital, but the same costs must be covered by the physician in an office, or non-facility, setting. These costs are also separately reimbursed by Medicare to the hospital/facility in addition to value of the procedure performed by the physician. Often, these facilities costs and fees reimbursed by Medicare greatly exceed the fee paid to the physician. Previous studies have shown discrepancies of up to 2156 % in sites of service procedures [17]. The data collected in this study shows that the unadjusted Medicare reimbursement rates (Fig. 1) and adjusted Medicare reimbursement rates in 2023 dollars (Fig. 2) both demonstrate downwards trends. However, both curves show that procedures performed in facility settings are reimbursed at lower rates compared to non-facility settings. It is important to note that the data collected and analyzed in this study solely represents Medicare reimbursement rates influenced by assigned RVUs and does not account for the overhead facility and non-facility fees and expenditures. Previous studies have shown that costs of the same procedure at facility sites of care were significantly higher than at non-facility sites [[18], [19], [20]].

The adjusted data shows no significant difference in change in total percent change or CAGR between the two data sets. However, there was a significant difference between the annual change. The adjusted average annual change decreased far more in non-facility (-$18.66) procedures than in facility (-$6.76) procedures (Table 5), showing that the reimbursement in dollar amount for the same procedures in a non-facility setting decreased significantly more per year than in facility settings. One possible explanation for the greater decrease in reimbursement rates in non-facility settings may be attributed to the overall higher average reimbursement amount in non-facility settings (Table 4). When all IP procedures were analyzed together, non-facility settings were found to have consistently higher reimbursement amounts than facility settings, which could explain the greater decrease in reimbursement rate per year. Another possible explanation to this trend is the shift in physician employment and practice acquisition by hospitals [[21], [22], [23]]. The overall reduction in reimbursement rates has driven procedures from a clinical, or non-facility setting, to hospital facility settings [24]. After the passage of the Affordable Care Act, the concept of Accountable Care Organizations encouraged providers unable to meet requirements for independent practices to merge with larger hospitals. This led to a decrease in non-facility locations and procedures over this study period. In the past, physicians were self-employed or part of small practices. However, in recent years, providers are unable to sustain themselves in independent practices and there has been a significant decrease in physicians in solo or two-physician practices [22]. The consolidation of practices is necessary for survival and practices are forced to merge with larger hospitals to stay afloat.

Although this study evaluates the trends in Medicare reimbursement in IP procedures, similar studies have evaluated trends in other fields of medicine. Medicare reimbursement rates were found to have been steadily decreasing from 2000 to 2018 by an average of 24.4 % for all general surgery procedures [3]. Between 2000 and 2016, reimbursement was also found to have steadily decreased in orthopedic procedures [4]. Between 2012 and 2015, total radiation treatments declined by about 7 % [5], and between 2007 and 2019, Medicare reimbursement rates declined across all diagnostic imaging modalities with few exceptions [6]. Current literature suggests that there is a consistent steady decrease of Medicare reimbursement across multiple studied fields, though more analysis is needed.

This study has possible limitations. The publicly available data utilized in this study was averaged nationally, and therefore the scope of analysis does not examine any relations between geographical areas and reimbursement trends. The study also does not show the proportion of practicing providers that are operating in facility or non-facility capacities, and how that proportion may vary by region. Instead, the data analysis is specifically representative of general, overall national trends.

## 5 Conclusion

After adjusting for inflation, the percent change in Medicare reimbursement rates were shown to have continuously decreased annually from 2000 to 2023 for the top 32 IP procedures in both non-facility (−60.40 %) and facility (−61.31 %) settings. However, this study showed that the average reimbursement rate for IP procedures done in non-facility (-$18.66) settings have significantly decreased more in US dollars ($) on an annual basis when compared to facility (-$6.76) settings. The findings of this study should support future legislative and administrative changes necessary to combat decreasing reimbursement rates as hospital expenditure and inflation continue to rise to ensure the continued success of patient care in this field. Further study and understanding of these trends will aid in discourse related to optimizing interventional pain care delivery and costs.

## Funding disclosures

No funding was received for this work.

## Declaration of competing interest

The authors declare that they have no known competing financial interests or personal relationships that could have appeared to influence the work reported in this paper.
